# Factors Associated with Risk of Stroke-Associated Pneumonia in Patients with Dysphagia: A Systematic Review

**DOI:** 10.1007/s00455-019-10061-6

**Published:** 2019-09-06

**Authors:** Sabrina A. Eltringham, Karen Kilner, Melanie Gee, Karen Sage, Ben D. Bray, Craig J. Smith, Sue Pownall

**Affiliations:** 1 Speech and Language Therapy Department, Sheffield Teaching Hospitals NHS Foundation Trust, Sheffield, UK; 2 Faculty of Health and Wellbeing, Sheffield Hallam University, Sheffield, UK; 3 King’s College London, London, UK; 4 Greater Manchester Comprehensive Stroke Centre, Manchester Academic Health Science Centre, Salford Royal Foundation Trust, Manchester, UK; 5 Division of Cardiovascular Sciences, University of Manchester, Manchester, UK

**Keywords:** Deglutition, Deglutition disorders, Dysphagia, Stroke-associated pneumonia, Stroke, Risk factors

## Abstract

Dysphagia is associated with increased risk of stroke-associated pneumonia (SAP). However, it is unclear what other factors contribute to that risk or which measures may reduce it. This systematic review aimed to provide evidence on interventions and care processes associated with SAP in patients with dysphagia. Studies were screened for inclusion if they included dysphagia only patients, dysphagia and non-dysphagia patients or unselected patients that included dysphagic patients and evaluated factors associated with a recorded frequency of SAP. Electronic databases were searched from inception to February 2017. Eligible studies were critically appraised. Heterogeneity was evaluated using *I*^2^. The primary outcome was SAP. Eleven studies were included. Sample sizes ranged from 60 to 1088 patients. There was heterogeneity in study design. Measures of immunodepression are associated with SAP in dysphagic patients. There is insufficient evidence to justify screening for aerobic Gram-negative bacteria. Prophylactic antibiotics did not prevent SAP and proton pump inhibitors may increase risk. Treatment with metoclopramide may reduce SAP risk. Evidence that nasogastric tube (NGT) placement increases risk of SAP is equivocal. A multidisciplinary team approach and instrumental assessment of swallowing may reduce risk of pneumonia. Patients with impaired mobility were associated with increased risk. Findings should be interpreted with caution given the number of studies, heterogeneity and descriptive analyses. Several medical interventions and care processes, which may reduce risk of SAP in patients with dysphagia, have been identified. Further research is needed to evaluate the role of these interventions and care processes in clinical practice.

**Electronic supplementary material:**

The online version of this article (10.1007/s00455-019-10061-6) contains supplementary material, which is available to authorized users.

## Introduction

Stroke-Associated Pneumonia (SAP) is common post stroke affecting 14% of patients [1], and is associated with increased risk of in hospital mortality [2], prolonged length of hospital stay [3], and has considerable economic impact on healthcare resources [4]. The pathophysiology of SAP is multifactorial. The combination of stroke-induced immunodeficiency and aspiration of oropharyngeal secretions and gastric contents into the lungs related to impaired consciousness and dysphagia predisposes patients to SAP in the first few days post stroke [5]. Respiratory tract infections may also precede stroke thereby contributing to stroke etiopathogenesis [6].

Acute stroke impairs the peripheral immune system, which is mediated by over-activation of the sympathetic nervous system and hypothalamic–pituitary–adrenal axis. Inhibition of peripheral cellular immune responses is characterized by transient lymphopenia and monocyte deactivation, which increases susceptibility to infection [7]. In a murine model of human stroke, stroke mice developed pan-lymphocytopenia and lymphocyte apoptosis in lymphoid tissues, which was reversed by either β-adrenergic receptor blockade or glucocorticoid receptor inhibition [8]. Alteration of tracheal epithelium caused by stroke immunomodulation has been shown to impair pulmonary clearance [9]. Reduced pulmonary clearance and impaired mobility related to decreased airway entry and impaired drainage of secretions from the lungs may contribute to development of pneumonia [9, 10].

Patients with dysphagia are more than three times at risk of developing pneumonia after stroke and the risk increases 11-fold in patients with confirmed aspiration [11]. Early dysphagia screening and specialist swallow assessment by a speech and language pathologist (SLP) may reduce the risk of SAP [12]. However, patients who are exclusively fed via the enteral route are also at risk of developing SAP. Tube feeding [13] and poor oral hygiene [14] may increase the risk of pneumonia by promoting bacterial colonization of the oropharynx. The presence of oral and dental disease causes alterations of oropharyngeal flora, and reduced saliva flow increases the bacterial density of the saliva. The presence of a nasogastric tube (NGT) may impact on bacterial colonization due to formation of biofilms on the tube [13], and predispose patients to gastro-esophageal reflux and vomiting [15]. Aspiration of bacteria laden secretions and infected refluxed material increases the risk of pneumonia. Functional status such as dependence for oral care and feeding has been shown to be significantly associated with respiratory infection [16].

A range of factors may be associated with SAP. These include risk factors associated with patient characteristics such as age, stroke severity, level of consciousness, as well as co morbidities such as chronic obstructive pulmonary disease and coronary artery disease [17]. However, these risk factors are outside the scope of this review. For this review, factors were defined as medical interventions to manage physiological status and care processes systemic to patients with dysphagia, in acute phase stroke and were identified from references and citation searching from a precursory systematic review [12].

The role of these pathophysiological processes in contributing to SAP in stroke patients with dysphagia, and the potential for therapeutic interventions to prevent SAP, is not well understood. We therefore undertook a systematic literature review with the aim of identifying care processes and/or interventions that were associated with modified risk of SAP in patients with dysphagia in acute stroke as targets for future clinical trials and evidence for implementation of a care process or intervention.

## Methods

### Search Strategy and Selection Criteria

A systematic review was undertaken according to the Preferred Reporting Items for Systematic Reviews and Meta-Analyses (PRISMA) statement [18], and Centre for Reviews and Dissemination guidance [19]. A building block [20] approach identified search terms for each concept. The concepts were dysphagia (Concept A), stroke (Concept B), risk factors (Concept C) and SAP (Concept D). These were combined using the Boolean AND operator. Two search strategies were used to develop the search terms: National Clinical Guideline for Stroke [21] and the Pneumonia in Stroke Consensus (PISCES) Group [22]. Co-authors (SP, KS, MG) reviewed the search strategy (Electronic Supplementary Material). Electronic databases were searched from inception to 14/2/2017 for relevant studies: CINAHL (via EBSCOhost), COCHRANE (via Wiley Online), EMBASE (via NICE Healthcare Databases), MEDLINE (via EBSCOhost) and SCOPUS. In addition, references and citations of included studies were screened. An example of the search strategy for the MEDLINE search is included in the Supplementary Material (Table1).

The review was restricted to peer-reviewed English language stroke research. Studies of dysphagia only patients, studies comparing dysphagia and non-dysphagia patients and unselected patients that reported dysphagia and evaluated factors associated with a recorded frequency of SAP were included. Acute phase stroke is typically defined as ≤ 72 h from admission. The time restriction of ≤ 72 h might not be explicit in the title/abstract; therefore, if the abstract met all the other inclusion criteria, it was included in the next stage of the screening process. Non-stroke or mixed population studies, those of exclusively intubated and mechanically ventilated patients, and studies not documenting SAP or pneumonia post stroke or pre-existing pneumonia were excluded.

Medical interventions included NGT feeding, oral care and prophylactic measures, for example, screening for immunodepression, antibiotics, management of gastro-esophageal reflux and the use of angiotensin-converting enzyme (ACE) inhibitors which have been suggested to reduce risk of pneumonia [23, 24]. Care processes included positioning, mobilization and staff competences and adherence to safe swallowing techniques. The primary outcome of interest was SAP. SAP is defined as the spectrum of lower respiratory tract infections within the first 7 days after stroke onset [22]. However, given the variation in reporting of post-stroke pneumonia and difficulty establishing stroke onset in some patients, for the purpose of this review studies were included that reported pneumonia within hospitalization and ≤ 30 days of stroke onset.

Two authors independently applied the inclusion/exclusion criteria to titles and abstracts for eligibility (Supplementary Material Table 2). Differences were forwarded to a third author for consensus. Abstracts that met the inclusion criteria were recommended for full-text reading and assessed by SAE. Corresponding authors were contacted to resolve eligibility and/or data extraction issues.

### Data Abstraction and Analysis

SAE designed and piloted a data extraction form based on Royal College of Physicians National Clinical Guideline for Stroke [25] and independently extracted data for the titles. Data extraction included study design, baseline characteristics of the population, factors and association with SAP (Supplementary Material Tables 3–4). Authors were contacted if data were not available. The extracted results were synthesized into the defined groups and organized thematically based on the National Clinical Guideline for Acute stroke care [21].

### Risk of Bias

Randomized control trials (RCTs) were assessed for risk of bias and quality [26]. Risk of bias tables were used to describe the methods used in each study and whether the results were at risk (Supplementary Material Table 5). Non-RCTs were assessed using the Critical Appraisal Skills Programme (CASP) checklists [27].

### Statistical Analysis

Inter-rater reliability for the inclusion/exclusion criteria was analysed using the Kappa statistic. The percentage of variation across studies due to heterogeneity was evaluated using I squared (*I*^2^) [28]. Review Manager 5.3 [29] and Microsoft Excel produced forest plots for illustration only [30].

## Results

**Fig. 1:**
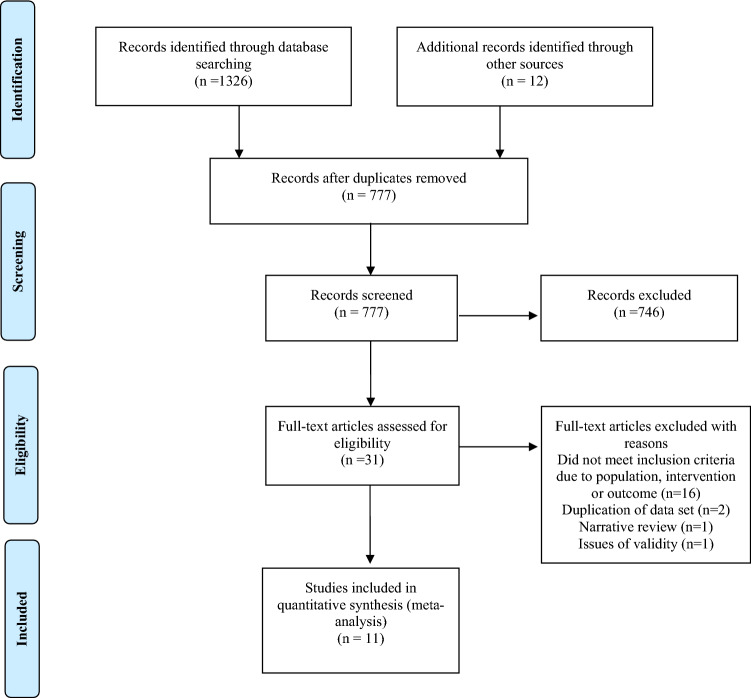
Search methodology and outcome

Database searching found 1326 references and 12 arose through other sources (Fig. 1). Inter-rater reliability for the inclusion/exclusion criteria was 0.78. Thirty-one full-text articles were assessed for eligibility. Eleven studies of 10 ischemic and hemorrhagic stroke patient cohorts were included (Table 1). Kalra et al. [31] and Kalra et al. [32] used the same RCT data. Study designs included RCTs (30%) [15, 31-33], prospective (20%) [13, 34] and retrospective (40%) [35-38] observational studies and one quasi-experimental design [39]. Europe hosted 55% of studies [15, 31–34, 37], Australia 27% [13, 36, 38] and Japan 18% [35, 39]. Five studies included dysphagia only populations [15, 31, 32, 35, 37], 2 studies included patients with and without dysphagia [33, 34], and 4 were unselected [13, 36, 38, 39]. There was variation in the way participant characteristics such as National Institutes of Health Stroke Scale (NIHSS) and age were reported and missing information. Based on available data, the overall mean NIHSS score was 12 [15, 31, 32, 34, 35, 37, 39] and mean age of participants was 76 years [15, 31, 32, 34, 35, 37-39].

**Table 1:** Study characteristics

Author, year, county	Study design	Stroke type	Participants	Intervention	Association with SAP
Aoki et al. (2016), Japan	Quasi-experimental	Ischemic and hemorrhagic	132 pre/173 post unselected; Age pre 70.0 ± 12.2 vs. post 70.1 ± 11.5 (*p* 0.91) Mdn NIHSS pre 5 (IQR 2–13) vs. 5 (IQR 2–14) post	MDT swallowing approach	aHR 0.41, 95% CI 0.19–0.84, *p* = 0.02
Arai et al. (2017), Japan	Retrospective observational	Ischemic and intracerebral hemorrhage	335 dysphagia only; Mdn age 82 years (IQR, 74–88 years) Mdn NIHSS 15 (11–24)	Histamine H2-Blocker or PPI or none	RR of H2B 1.24, 95% CI; 0.85–1.81 and 2.00 in PPI, 95% CI 1.12–3.57
Brogan et al. (2015), Australia	Retrospective observational	Unreported	533 unselected; Age > 80 years. 33.4%	NGT	OR 3.91; 95% CI 1.73–8.80; *p* = 0001
Gandolfi et al. (2014), Italy	Retrospective observational	Ischemic and hemorrhage	84 dysphagia only; 39T+ vs. 45T− M (± SD) age 77.9 (8.55). NIHSS 13.88 (7.12)	MDT swallowing approach	aOR 0.34, 95% CI 0.07–1.49
Gosney et al. (2006), UK	RCT double-blind PBO	Unreported	203 (58 w/dysphagia); Mdn age: active 78 years vs. placebo 62 years. (H1), active 68 years vs. PBO 74 years. (H2), active 71 years vs. PBO 74 years (H3)	SDD oral gel	7/8 dysphagia patients developed pneumonia (*N* = 1 active vs. 6 placebo)
Hoffman et al. (2016), Germany	Prospective observational	Ischemic	484 (111 w/ dysphagia); *M* age 69.9 (11.8). Mdn (IQR) NIHSS 4 (2–7)	Screening for SAP, dysphagia and biomarkers	Dysphagia and decreased monocytic HLA-DR predictors of SAP
Kalra et al. (2015), UK	Prospective, multicenter, cluster RCT	Ischemic and hemorrhagic	1088 dysphagia only; *M* age (SD) 77.8 (12.0), Mdn NIHSS 15 (IQR 9–20)	Prophylactic Antibiotics	Algorithm SAP; aOR 1.21; 95% CI 0.71–2.08, *p* = 0.489
Kalra et al. (2016), UK	Prospective, multicenter, cluster RCT	Ischemic and hemorrhagic	1088 dysphagia only; *M* age (SD) 77.8 (12.0), Mdn NIHSS 15 (IQR 9–20)	NGT	Algorithm SAP; aOR 1.26, 95% CI 0.78–2.03, *p* = 0.353
Langdon et al. (2009), Australia	Prospective observational	Ischemic	330 unselected; *M* age SAP (SD) 71.7 ± 13.0	NGT	aRR 2.76 (95% CI 1.26–6.01), *p* = 0.011
Schwarz et al. (2017), Australia	Retrospective cohort	Ischemic	110 unselected, Ave age 69.87, range 28–94	NGT	RR 12.609 (95% CI, OR 21.54), *p* < 0.0001
Warusevitaine et al. (2014), UK	Phase II RCT double-blind PBO	Ischemic and hemorrhagic	60 dysphagia only, *M* age 78. M NIHSS 19.25	Metoclopramide	aRR 5.24 (95% CI 2.43–11.27), *p* value < 0.001

### Assessment of Quality And Bias

Study quality ranged from high-quality RCTs to moderate quality quasi-experimental studies to lower quality retrospective observational studies (Supplementary Material Table 4). Overall, the RCTs were deemed to have a low risk of bias. Potential sources of selection bias in the cluster RCT studies [31, 32] included where patients at increased risk of SAP might have been preferentially recruited into the intervention group. A limitation of the Kalra et al. [31] study was that data were derived from an RCT and a prospective cohort data structure was assumed, which may have resulted in selection bias. A possible source of performance bias was participants and researchers being aware of allocation treatment. The open intervention allocation could potentially influence physician diagnosis of pneumonia.

Other possible sources of bias and quality considerations in the RCT and non-RCT studies include small population size and risk of measurement bias. There was a lack of objective measurement of the MDT swallowing approach [39] and the potential bias of progressive proficiency of implementing the MDT protocol over time [37]. Other examples of measurement bias included lack of information about the diagnosis and method of assessment of dysphagia and subsequent measurement and severity rating, and classification of stroke severity.

### Diagnosis and Frequency of SAP

Overall incidence was reported in 10 studies [13, 15, 31-36, 38, 39] (Supplementary Material Table 5, Fig. 1) and ranged from 3.9 to 56.7% [15, 33], with the largest dataset at 11.3% [31, 32]. The Centers for Disease Control and Prevention (CDC) criteria [40] were used to define pneumonia in the majority of studies. One study made a diagnosis based on the British Thoracic Society recommendations [15]. The STROKE-INF trial data set used blinded application of CDC criteria and physician-diagnosed pneumonia [31, 32]. Four used a combination of clinical symptoms, radiologic findings on X-ray and laboratory results and combined antibiotics [13, 15, 33, 37]. Two studies provided no definition [36, 38].

Measurement of pneumonia timing varied. Four studies reported pneumonia during hospitalization [33, 34, 37, 39]. Three studies reported within 14 days of admission [31, 32, 35] and one from 7 days of admission [36]. Warusevitaine et al. [15] and Langdon et al. [13] reported at 21 days and 30 days, respectively. Schwarz et al. [38] did not report the period of diagnosis. Marked variation in study design and reporting of participant characteristics prohibited meta-analysis.

### Medical interventions

#### Prophylactic Measures

##### Screening for Stroke-Induced Immunodepression

One study [34] investigated the predictive properties of biomarkers of immunodepression (mHLA-DR expression), as well as inflammation (IL-6), and infection (LBP) during the acute phase of stroke, and incidence of SAP stratified for patients with and without dysphagia.

*Incidence and risk of SAP* Incidence of SAP in patients with dysphagia was 16.2% vs. 5.2% overall. When combining all three biomarkers and presence of dysphagia, only mHLA-DR [OR 0.29 (95% CI 0.09–0.94; *p* = 0.0398)] and dysphagia [OR 5.74 (95% CI 2.21–14.89; *p* = 0.0003)] were independent predictors of SAP. Patients with dysphagia and low mHLA-DR expression were at particularly high risk of SAP (18.8%). In patients without dysphagia and who had normal mHLA-DR expression, no SAP was observed (0%).

##### Medication Use

Four studies investigated use of pharmacological agents for reducing pneumonia: prophylactic antibiotics [32], acid suppressive medications [35], metoclopramide—an antiemetic and prokinetic drug [15], and selective decontamination of the digestive tract (SDD) [33]. No studies assessed ACE inhibitors and their association with SAP in patients with dysphagia. Three studies were RCTs [15, 32, 33]. Preventative antibiotics were administered in Nil by mouth (NBM) patients ≤ 48 h post onset of stroke symptoms [32]. In a second study, patients who were unable to eat orally for 14 days or more after admission were exposed to acid suppressive drugs: famotidine, a Histamine H2-Blocker (H2B), and omeprazole, a Proton Pump Inhibitor (PPI) [35]. The choice of drugs was at the discretion of the treating physician. Warusevitaine et al. [15] study participants received metoclopramide or placebo 3× daily via the NGT for 21 days or until NGT feeds were discontinued. SDD involved oral gel containing antimicrobial drugs, applied topically to the mouth four times daily. Patients were randomized to receive either the SDD gel or placebo. Treatment was continued for 3 weeks for patients with dysphagia and for 2 weeks for those with a normal swallow.

*Incidence and risk of SAP* Kalra et al. [32] found that prophylactic antibiotics did not affect the incidence of algorithm-defined post-stroke pneumonia in the antibiotic group (13%) versus the control group (10%) (aOR 1.21; 95% CI 0.71–2.08, *p* = 0.489). Additionally, no differences were noted in physician-diagnosed post-stroke pneumonia between dysphagic patients in the antibiotic group (16%) versus the control group (15%) (aOR 1.01; 95% CI 0.61–1.68, *p* = 0.957).

Arai et al. [35] found that the daily incidence of pneumonia in the PPI group (6.38%, 95% CI 3.78–10.1) was 1.7 times higher than in the exposed H2B group (3.77%, 95% CI 2.92–4.78). PPI use in patients with dysphagia was associated with increased risk of pneumonia (RR 2.00, 95% CI 1.12–3.57), while use of H2B was not (RR 1.24, 95% CI 0.85–1.81).

Warusevitane et al. [15] found there were significantly more episodes of pneumonia in the placebo group (RR 5.24, 95% CI 2.43–11.27; *p* < 0.001) than the metoclopramide group: placebo group mean 1.33 (SD 0.76) vs. metoclopramide group mean 0.27 (SD 0.45).

In Gosney et al. [33], 3.94% (*N* = 8) patients developed pneumonia. Seven of the 8 cases of pneumonia occurred in patients with dysphagia. Patients with dysphagia were twice as likely to have AGNB (aerobic Gram-negative bacteria) organisms, which are implicated in aspiration pneumonia, present in their first swab (< 24 h of admission) than those with a normal swallow, although this did not reach significance. Only 1 dysphagic patient treated with SDD developed pneumonia compared to 6 dysphagic patients in the placebo group. The study did not provide data on how many dysphagic patients with AGNB developed pneumonia compared to those with dysphagia without AGNB.

##### Nasogastric Tubes (NGTs)

Four studies [13, 31, 36, 38] investigated association between NGTs and SAP in acute stroke patients. The characteristics of these studies varied between unselected patients that included patients with dysphagia [13, 36, 38] and dysphagia only patients [31]. Kalra et al. [31] used the STROKE-INF data set where patients had been randomly assigned to be given either prophylactic antibiotics or standard stroke unit care. Three studies provided experimental and control data [13, 31, 36].

*Incidence and risk of SAP* Overall incidence of SAP varied between and within studies. Brogan et al. [36] (37%) and Langdon et al. [13] (41%) reported higher incidence of SAP compared to Kalra et al. who reported rates of incidence for physician-diagnosed (18.5% vs. 15.3%, *p* = 0.21) and algorithm-defined SAP in NGT-fed and No-NGT patients (14.4% vs. 10.1%, *p* = 0.046). The higher rate of algorithm SAP in patients with NGT did not remain significant after adjustment for age, stroke type, severity and chronic lung disease (aOR 1.26; 95% CI 0.78–2.03, *p* = 0.35). Patients with NGT had more severe strokes with impaired consciousness. Preventive antibiotics did not reduce incidence of SAP in patients with NGT [aOR 1.05 (95% CI 0.73–1.52); *p* = 0.803]. Schwartz et al. [38] did not report incidence of SAP in patients with NGT and did not respond to information requests by the author. Differences in SAP incidence between studies can be partly explained by the different study populations and the lack of adjustment for stroke severity and baseline characteristics [13, 36].

There was a high degree of heterogeneity between the three studies (*I*^2^ = 94%) [13, 31, 36] that provided experimental (NGT) vs. control (No NGT) data. The incompatibility of study designs precluded presenting the data as a meta-analysis. Based on the individual studies, Kalra et al. found no evidence that NGT increased SAP (aOR 1.26; 95% CI 0.78–2.03, *p* = 0.35). In contrast, Brogan et al. found having an NGT (OR 3.91; 95% CI 1.73–8.80; *p* = 0001) and being NBM (OR 5.62; 95% CI 1.54–20.46; *p* = 0.0089) were independently associated with respiratory infections. Langdon et al. also found being enteral fed during admission was a significant risk factor for respiratory infection (aRR 2.76; 95% CI 1.26–6.01, *p* value 0.011). Schwarz et al. found the presence of an NGT significantly increased the risk of developing aspiration pneumonia (*p* < 0.0001) with a relative risk of 12.609 (95% CI, OR 21.54).

### Care Processes

#### Multidisciplinary Team Approach (MDT) To Swallowing

Two studies described the implementation of a MDT approach to dysphagia, in dysphagia only [37] and unselected patients [39]. Aoki et al. MDT participatory team comprised of 9 professionals including doctors, dentists, nurses, physiotherapists (PT), occupational therapists (OT), SLPs, managerial dieticians, dental hygienists and pharmacists. The approach was the cooperation of the various professionals that have the skills to improve the quality of medical care, utilizing the specialist knowledge and skills of each professional. To understand the difference of the MDT approach, frequencies of professional oral care and swallowing evaluations before team organization (‘prior period’) and the period after team organization (‘post period’) were evaluated.

In Gandolfi et al. [37], a standardized diagnostic and rehabilitative protocol for stroke related dysphagia management was progressively introduced. A MDT of neurologists, nurses, rehabilitation physicians, PTs, nutritionist, SLPs, radiologists and ear nose throat specialists were involved in the implementation. The protocol consisted of 2 phases: a diagnostic phase, aiming to define the swallowing problem and selecting those patients who were eligible for the following rehabilitative phase. The diagnostic phase included clinical and instrumental evaluation by fiber-optic endoscopic evaluation (FEES) and/or videofluoroscopy (VFSS). Rehabilitative treatment for dysphagia proceeded in 3 consecutive phases: Phase 1 sensory stimulation of the oral cavity, oro-facial and breathing exercises, Phase 2 swallowing trials of crushed iced and jellied water and teaching airway protection strategies and Phase 3 weaning from nutritional support by administration of small semisolid meals fractionated throughout the day. During hospitalization the patients received 1-hour individual sessions of rehabilitation for dysphagia. Pneumonia rates were compared after pre implementation of the protocol for dysphagia (T− group) versus after the implementation of the MDT protocol (T+  group).

##### Incidence and Risk of SAP

Aoki et al. found pneumonia onset was less frequent in the post group compared to the prior group (6.9% vs. 15.9%; *p* = 0.01) and a MDT swallowing approach was related to reduced occurrence of pneumonia onset independent of NIHSS score on admission (aHR 0.41, 95% CI 0.19–0.84, *p* = 0.02). The percentage of patients receiving professional oral care (51.7% vs. 12.9%, *p* < 0.0001) and instrumental swallowing evaluations (26.0% vs. 12.1%, *p* = 0.002) were significantly increased in the post group. Gandolfi et al. reported no significant differences between the two groups in the frequency of pneumonia but did not provide incidence data. There was very weak evidence of a reduction in pneumonia risk for the T+ group [aOR 0.34 (0.07–1.49)] compared to the T− group.

#### Mobility

Two studies, both of unselected patients investigated reduced mobility and the impact on SAP [13, 36].

##### Incidence and Risk of SAP

Both studies found patients who required full assistance with mobility or had impaired mobility on admission were at significant risk of SAP. Brogan et al. [36] found odds of infection were 6.48 times (95% CI 1.35–31.16;* p* = 0.0198) for patients who required full assistance with mobility than those who were able to mobilize. Langdon et al. found impaired mobility on admission was a significant risk factor for respiratory infection (aRR 2.86; 95% CI 1.26–6.48, *p* value 0.012) [13].

#### Other Care Processes

No studies were retrieved from the search strategy relating to positioning or adherence with recommendations from the dysphagia screen or specialist swallow assessment.

## Discussion

We have identified a range of medical interventions and care processes, which may impact on the development of SAP in patients with dysphagia. However, there are insufficient data to recommend any of these at present and interpretation is limited by heterogeneity of studies and reporting. This review has identified a need for further research of candidate processes and interventions.

There is emerging evidence for the use of preventative measures such as screening for stroke-induced immunosuppression and considering instrumental swallow assessment in patients with low mHLA-DR expressions who have been identified with dysphagia. Further RCTs with larger sample sizes are needed to test this hypothesis and screening for AGNB organisms. Studies need to evaluate the utility and external validity of these medical interventions specifically in relation to optimal timing, point-of-care technology, and what they add to existing dysphagia assessment methods. Further research is also required to evaluate what the intervention might be, for example boosting the immune system in the acute phase, or treating with SDD gel for the duration of the patients' dysphagia.

The findings of Kalra et al. [32] are consistent with a recent Cochrane Review [41] which found high-quality evidence that antibiotic prophylaxis in people with acute stroke does not reduce post-stroke pneumonia (RR 0.95, 95% CI 0.80–1.13). The PRECIOUS (PREvention of Complications to Improve OUtcome in elderly patients with acute Stroke) Trial is assessing if metoclopramide prevents aspiration [42]. This has the potential to inform whether the use of metoclopramide can reduce risk of pneumonia shown by Warusevitaine et al. The one study included in this review found that PPI use in non-orally fed patients was significantly associated with increased risk of pneumonia while H2B was not, suggesting PPI may have to be avoided in those at high risk for pneumonia. There is equivocal evidence that NGT placement increases risk of SAP due to high degree of heterogeneity between studies. Further studies are needed to evaluate if treatment with H2B and PPI, and NGT use are implicated in the risk of SAP in patients with severe dysphagia.

A number of studies support the argument for a critical period of susceptibility for post-stroke infection [13, 15, 34, 36]. Warusevitaine et al. found of the patients that developed pneumonia, for 94% of patients this occurred within 7 days post admission; the mean time from NGT insertion to the first episode of pneumonia was 4 days in the treatment group and 2 days in the placebo group. Langdon et al. propose to hold off institutional enteral feeding for the first 3–4 days concentrating on maintaining hydration via intravenous or sub-cutaneous methods suggesting this may reduce the risk of post-stroke infection from stroke-induced immunodeficiency and allow spontaneous recovery of swallow function.

Both studies evaluating a MDT approach [37, 39] to swallowing management found this impacted positively on reducing risk of incidence of SAP. This supports previous studies that have demonstrated an integrated team approach and dysphagia clinical pathway has a positive impact on rates of pneumonia [43-47]. However, Aoki et al. lacked clarity about what the intervention involved. Improvement in pneumonia rates was attributed to increased oral care by dental professionals and instrumental assessments by SLPs, and the creation of appropriate dysphagia diets and nutritional supplements by dieticians. Similarly Gandolfi et al. lacked detail about what components of the intervention had a positive impact on patient outcomes. Both studies used either FEES and/or VFSS instrumental assessments and emphasized the cooperation and utilization of different professionals. Additionally, the inclusion of an evaluation of postural control by Gandolfi et al. may have been a contributory factor to the success of the MDT management. However, it might be argued that in the Gandolfi study, dysphagia received greater attention in the T+ group with the implementation of the specific protocol rather than the protocol itself. The study also did not necessarily apply typical care routines within their teams, for example the rehabilitation physician rather than the SLP undertook the clinical bedside swallow assessment.

This review acknowledges certain limitations. There is a risk of selection bias. Studies were identified based on the selection criteria. We acknowledge that there are other studies that include dysphagic patients within unselected trial populations but because they did not report data specifically for this population, they were not retrieved by our search. For example, Anderson et al. [48] examined whether lying flat versus sitting up at least 30 degrees as an early intervention in stroke care would improve outcomes in patients with ischemic stroke. There was no difference between the two groups in mortality (7.3% lying flat vs. 7.4% sitting up) or major disability (mRS 4–6) (38.9% lying flat vs. 39.7% sitting up). There was no significant between-group difference in the rate of pneumonia. However, data for patients with dysphagia were not reported which meant that this study would not have been retrieved by the search strategy. In this study, patients with a definite clinical indication or contraindication of being laid flat were excluded, such that patients with severe dysphagia may have been excluded. Other examples of selection bias were that only a small number of studies were identified which met the inclusion criteria for each factor and in some cases no relevant studies were found.

The pathoetiology of SAP is a combination of stroke-induced suppression of immune responses and pulmonary infectious challenge as a consequence of aspiration of oropharyngeal secretions and gastric contents into the lungs in the first few days post stroke. The Pneumonia in Stroke Consensus (PIECES) group defines SAP as a spectrum of lower respiratory infections within the first 7 days after stroke onset and diagnosis of SAP are based on the Centers for Disease Control and Prevention (CDC) criteria [22]. Examples of reporting bias include variation in the diagnostic criteria for SAP and the period of diagnosis in the included studies. There may also be the possibility that non-infective causes of lung inflammation (e.g. pneumonitis) may have been reported as pneumonia. Further examples of reporting bias include the lack of information on the diagnosis and method of assessment of dysphagia and measure of severity. Therefore, the findings need to be interpreted with caution.

A further limitation was the sole use of British orthography for terms “oesophageal” and “GORD”. This may have precluded identification of some records using the American orthography. The use of the MeSH term “deglutition disorders” should have limited the impact of this omission.

## Conclusion

This review has shown SAP is associated with a range of interventions and care processes and there is increased susceptibility in the acute phase for patients with dysphagia. Measures of immunodepression are associated with SAP in dysphagic patients. However, there is insufficient evidence to suggest screening for immunosuppression at this stage. There is absence of evidence that prophylactic antibiotics make a difference to pneumonia rates in patients with dysphagia and use of PPIs may be associated with increased risk. There is insufficient evidence to justify screening for aerobic Gram-negative bacteria. Treatment with metoclopramide may reduce SAP risk. A multidisciplinary team approach and instrumental assessment of swallowing may reduce risk of pneumonia. The evidence that NGT placement increases risk of SAP is equivocal. Impaired mobility is associated with increased risk. Further studies should examine these factors and the potential to reduce the incidence of SAP in patients with dysphagia using instrumental methods of assessment and standardized measurement criteria.

## Electronic supplementary material

Below is the link to the electronic supplementary material.

Supplementary file1 (DOCX 131 kb)
