# Supplementary material for: Factors Associated with Risk of Stroke-Associated Pneumonia in Patients with Dysphagia: A Systematic Review
Source: Dysphagia. 2019 Sep 6;35(5):735–44. doi: 10.1007/s00455-019-10061-6 (PMC7522065; doi:10.1007/s00455-019-10061-6)
Supplement: Supplementary file 1 — Supplementary file1 (DOCX 131 kb) [file 455_2019_10061_MOESM1_ESM.docx]

**ELECTRONIC SUPPLEMENTARY MATERIAL**

**Factors associated with risk of stroke-associated pneumonia in patients with dysphagia: A Systematic Review**

Sabrina A. Eltringham, MSc;^1,2^ Karen Kilner, Dr.; ^2^ Melanie Gee, MA (Oxon), MSc; ^2^ Karen Sage, Prof.; ^2^ Ben. D. Bray, Dr;^3^ Craig.J.Smith, Prof; ^4,5^ Sue Pownall, Prof.^1;2^

^1^Speech and Language Therapy Department, Sheffield Teaching Hospitals NHS Foundation Trust, Sheffield, UK

^2^Faculty of Health and Wellbeing, Sheffield Hallam University, Sheffield, UK

^3^King’s College London, London, UK

^4^Greater Manchester Comprehensive Stroke Centre, Manchester Academic Health Science Centre, Salford Royal Foundation Trust, UK

^5^Division of Cardiovascular Sciences, University of Manchester, Manchester, UK

**Corresponding author** – Sabrina A. Eltringham, Tel: +44 114 271 2676, Fax: +44 114 271 3189, Email: s.a.eltringham@shu.ac.uk

**Itemized list of figures and tables:**

Table 1: Medline Search Strategy

Table 2: Inclusion/Exclusion Criteria

Table 3: Study Characteristics

Table 4: Criteria for SAP, period of diagnosis and incidence

Table 5: Quality Appraisal Tables

Figure 2: Overall rate of SAP

**Table 1: Medline Search Strategy**

S29 S3 AND S8 AND S27 AND S28

S28 S9 OR S10 OR S11 OR S12 OR S13 OR S16 OR S19 OR S20 OR S21

S27 S22 OR S23 OR S24 OR S25 OR S26

S26 (MH "Deglutition Disorders+")

S25 AB aphag* OR TI aphag* OR MW aphag*

S24 AB dysphag* OR TI dysphag* OR MW dysphag*

S23 AB ( (( swallow* n1(impairment or disorder or problem or difficulty) )) OR TI ( (( swallow* n1(impairment or disorder or problem or difficulty) ) ) OR MW ( (( swallow* n1 (impairment or disorder or problem or difficulty) ) )

S22 AB ( (( deglutition n1 (impairment or disorder or problem or difficulty) ) ) OR TI ( (( deglutition n1 (impairment or disorder or problem or difficulty) ) ) OR MW ( (( deglutition n1 (impairment or disorder or problem or difficulty) ) )

S21 AB ( GORD or "gastro oesophageal reflux disease" ) OR TI ( GORD or "gastro oesophageal reflux disease" ) OR MW ( GORD or "gastro oesophageal reflux disease" )

S20 TI ( (("angiotensinconverting enzyme" or ACE) n1 inhibitor*)) or ("histamine 2 receptor antagonist*" or "H2 blockers") ) OR AB ( (("angiotensinconverting enzyme" or ACE) n1 inhibitor*)) or ("histamine 2 receptor antagonist*" or "H2 blockers") ) OR MW (

(("angiotensinconverting enzyme" or ACE) n1 inhibitor*)) or ("histamine 2 receptor antagonist*" or "H2 blockers") )

S19 S17 OR S18

S18 AB ( "antibiotic* therapy" or "infection prevent*" or "prevent* antibiotic*" ) OR TI ( "antibiotic* therapy" or "infection prevent*" or "prevent* antibiotic*" ) OR MW( "antibiotic* therapy" or "infection prevent*" or "prevent* antibiotic*" )

S17 (MH "Antibiotic Prophylaxis")

S16 S14 AND S15

S15 AB ( ( (assess* or screen* or protocol* or test or manage* or recommend* or intervention) n3 (dysphag* or aphag* or swallow* or deglutition) ) ) OR TI ( ( (assess* or screen* or protocol* or test or manage* or recommend* or intervention) n3 (dysphag* or aphag* or swallow* or deglutition) ) ) OR MW ( ( (assess* or screen* or protocol* or test or manage* or recommend* or intervention) n3 (dysphag* or aphag* or swallow* or deglutition) ) )

S14 AB ( ( concordan* or adher* or "non adher*" or complian* or "non complian*" or cooperat* or co-operat* or uncooperat* or uncooperat* or engage* or disengag* or behaviour or behavior) n3 (assess* OR screen* OR protocol OR test OR manage* OR recommend* OR

intervention OR dysphag* OR aphag* OR swallow* OR deglutition) ) OR TI ( ( concordan* or adher* or "non adher*" or complian* or "non complian*" or cooperat* or co-operat* or uncooperat* or uncooperat* or engage* or disengag* or behaviour or behavior) n3 (assess* OR screen* OR protocol OR test OR manage* OR recommend* OR intervention OR dysphag* OR aphag* OR swallow* OR deglutition) ) OR MW ( ( concordan* or adher* or "non adher*" or complian* or "non complian*" or cooperat* or co-operat* or uncooperat* or uncooperat* or engage* or disengag* or behaviour or behavior) n3 (assess* OR screen* OR protocol OR test OR manage* OR recommend* OR intervention OR dysphag* OR aphag* OR swallow* OR deglutition) )

S13 AB ( educat* or train* or competen* or skill* or capabilit* or know* or abilit* or proficien* or exper* ) OR TI ( educat* or train* or competen* or skill* or capabilit* or know* or abilit* or proficien* or exper* ) OR MW ( educat* or train* or competen* or

skill* or capabilit* or know* or abilit* or proficien* or exper* )

S12 AB ( mobil* or immobil* or move* or activ* or inactiv* or rotat* or turn* ) OR TI ( mobil* or immobil* or move* or activ* or inactiv* or rotat* or turn* ) OR MW ( mobil* or immobil* or move* or activ* or inactiv* or rotat* or turn* )

S11 AB ( position* or post* ) OR TI ( position* orpost* ) OR MW ( Search Screen - Advanced

position* or post* )

S10 AB ( "nasogastric tube" or NGT or "feeding tube" or ((enteral or alternative) n1 (nutrition or feeding)) ) OR TI ( "nasogastric tube" or NGT or "feeding tube" or ((enteral or alternative) n1 (nutrition or feeding)) ) OR MW ( "nasogastric tube" or NGT or "feeding tube" or ((enteral or alternative) n1 (nutrition or feeding)) )

S9 AB (( oral or mouth or dental) n1 ( care or hygiene) ) OR TI ( (oral or mouth or dental) n1 (care or hygiene )) OR MW (( oral or mouth or dental) n1 (care or hygiene ))

S8 S4 OR S5 OR S6 OR S7

S7 AB ( (cerebro* or brain or brainstem or cerebral*)n3 (infarct* or accident*) ) OR TI (

(cerebro* or brain or brainstem or cerebral*)n3 (infarct* or accident*) ) OR MW ( (cerebro* or brain or brainstem or cerebral*)n3 (infarct* or accident*) )

S6 AB ( stroke* or CVA or "cerebrovascular accident" or "brain attack*" or "cerebral haemorrhage" or "cerebral hemorrhage" ) OR TI ( stroke* or CVA or "cerebrovascular accident" or "brain attack*" or "cerebral haemorrhage" or "cerebral hemorrhage" ) OR MW ( stroke* or CVA or "cerebrovascular accident" or "brain attack*" or "cerebral haemorrhage" or "cerebral hemorrhage" )

S5 (MH "Cerebral Hemorrhage+")

S4 (MH "Stroke+")

S3 S1 OR S2

S2 AB ( "stroke associated pneumonia" or "chest infection*" or "hospital acquired pneumonia" or "aspiration pneumonia" or "post stroke pneumonia" ) OR TI ( "stroke associated pneumonia" or "chest infection*" or "hospital acquired pneumonia" or

"aspiration pneumonia" or "post stroke pneumonia" ) OR MW ( "stroke associated pneumonia" or "chest infection*" or "hospital acquired pneumonia" or "aspiration pneumonia" or "post stroke pneumonia" )

S1 (MH "Pneumonia+")

Database - MEDLINE

Table 2: Inclusion/Exclusion Criteria

| **Inclusion Criteria** | **Exclusion Criteria** |
| --- | --- |
| Adults with dysphagia secondary to acute stroke | Mixed population studies, adults with pre existing dysphagia. Intubated or ventilated CVA patients. |
| Acute stroke i.e. Treated ≤ 72 hours of admission – acknowledgement that ≤ 72 hours of admission may not be explicit in abstract therefore should be included to screening full text if abstract refers to acute stroke. | > 72 hours of admission |
| Organisational factors: 1) oral hygiene, (2) nasogastric tube placement, (3) specific medication use e.g. for reflux management and cardiovascular disease, (4) positioning (for oral and enteral feeding, and to reduce reflux), (5) mobilisation, (6) adherence (to recommendations from the dysphagia ax) and (7) staff competencies in dysphagia | Non-systemic factors that are not related to managing risk of SAP in dysphagia stroke patients. |
| Stroke associated pneumonia or documentation of pneumonia after stroke onset. | Studies not documenting SAP or pneumonia post stroke. Pre existing pneumonia. |
| Peer reviewed, quantitative, qualitative and mixed method studies.  Primary data  Studies in Systematic reviews which meet inclusion criteria | Non-peer reviewed studies, grey literature, editorial letters, book reviews.  Conference abstracts/presentations.  Foreign language text where no English translation is available. |

Table 3 – Study characteristics

| Author, year, county | Study design | Participants | Intervention | Association with SAP |
| --- | --- | --- | --- | --- |
| Gosney et al. (2006), UK | Prospective, randomized, placebo-controlled double blind trial | 203 acute stroke patients; median age: active 78 yrs. vs. placebo 62 yrs. (Hospital 1), active 68 yrs. vs. placebo 74 yrs. (Hospital 2), active 71 yrs. vs. placebo 74 yrs. (Hospital 3), NIHSS not reported | SDD oral gel or placebo | 7/8 dysphagia patients developed pneumonia (N=1 active vs. 6 placebo). |
| Kalra et al. (2015), UK | Prospective, multicenter, cluster-randomized controlled trial | 1217 ischemic and hemorrhagic dysphagia patients clustered within 37 stroke units to two treatment groups: 615 antibiotic group, mean age (SD) 77.7 (11.9), median NIHSS 15 (IQR 9-20) vs. 602 in control group, age 78.0 (12.2), median NIHSS 14 (IQR 9-20) | Antibiotics initiated within 48h of symptom onset at intervention centers for 7 days plus standard care vs. standard care only | Algorithm defined SAP 13% antibiotic group vs. 10% SAP control group (aOR 1.21;95% CI 0.71-2.08, p=0.489). |
| Kalra et al. (2016), UK | Prospective, multicenter, cluster-randomized controlled trial | 1217 ischemic and hemorrhagic dysphagia patients: 298 NGT ≤48 hrs. onset vs. 790 without NGT; mean (SD) age 78.8 (11.8) NGT vs. 77.5 (12.1) without, median (IQR) NIHSS 17 (12-21) NGT vs. 14 (8-20) without | NGT placement within 48 hrs | NGT did not increase algorithm defined SAP (14.4% in NGT vs. 10.1% without), adjusted OR 1.26, (95% CI 0.78-2.03, p=0.353) |
| Warusevitaine et al. (2014) | Randomized double-blind placebo controlled phase II trial | 60 Acute stroke patients with dysphagia fed via NGT; N=30 Metoclopramide vs. N=30 Placebo; mean age (SD) Metoclopramide 76.9 (6.3) vs. 79.2 (10.8) Placebo, p value 0.4, mean NIHSS (SD) Metoclopramide 19.93 (5.96) vs. 18.57 (6.71) Placebo, p value=0.1. | Metoclopramide vs. placebo via NGT | Mean (SD) number of episodes of pneumonia 1.33 (0.76) control group vs. 0.27 (0.45) metoclopramide (aRR 5.24 (95% CI; 2.43-11.27), p value <0.001). |
| Aoki et al. (2016), Japan | Quasi-experimental | 132 acute stroke prior period/173 post period; prior age 70.0±12.2 vs. post 70.1±11.5 (p-value 0.91), median NIHSS prior 5 (IQR 2-13) vs. 5 (IQR 2-14) post. | MDT participatory swallowing team. | MDT swallowing approach was related to pneumonia onset independent of NIHSS score (aHR 0.41,95% CI 0.19-0.84, p=0.02). |
| Arai et al. (2017), Japan | Retrospective observational | 335 ischemic and intracerebral hemorrhage dysphagia patients; median age 82 yrs. (IQR, 74-88 yrs.); median NIHSS 15 (11-24) | Histamine H2-Blocker or PPI or none | RR by multivariate analyses was 1.24 in H2B (95% CI;0.85-1.81) and 2.00 in PPI (95%;1.12-3.57) |
| Brogan et al. (2015), Australia | Retrospective observational | 533 stroke patients. Age >80 yrs. 33.4%, NIHSS not reported. | Relationship between clinical factors and infections analysed using univariate and multivariate models. | NGT (OR 3.91;95% CI 1.73-8.80; p=0001) and being NBM (OR 5.62;95% CI 1.54-20.46;p=0.0089) were independently associated with respiratory infections. |
| Gandolfi et al. (2014), Italy | Retrospective observational | 84 ischemic and hemorrhage dysphagia patients. 39 treated group (T+) vs. 45 non treated group (T-); mean (± SD) age T+ 76.15 yrs. (9.70) vs. 79.53 yrs. (7.17), mean (± SD) NIHSS T+13.53 (7.12) vs. T- 14.21 (7.19) | MDT protocol for management of post stroke dysphagia | Significantly lower risk of pneumonia (aOR 0.34 [95% CI 0.07-1.49]) for T+ group vs. T- group. |
| Hoffman et al. (2016), Germany | Prospective observational | 484 Ischemic stroke; median (IQR) age 72 (62-78) yrs, median (IQR) NIHSS 4 (2-7). | Screening for SAP, dysphagia and biomarkers | Dysphagia and decreased monocytic HLA-DR independent predictors of SAP. |
| Langdon et al. (2009), Australia | Prospective observational | 330 ischemic stroke patients: 51 respiratory infection, mean age of patients with SAP (SD) 71.7± 13.0, NIHSS not reported. | Incidence of respiratory infection in NBM tube fed patients. | Enteral feeding during admission was a significant risk factor for respiratory infection during admission aRR 2.76 (95% CI 1.26-6.01), p=0.011 |
| Schwarz et al. (2017), Australia | Retrospective cohort | 110 ischemic stroke patients, average age 69.87, range 28-94. NIHSS not reported. | Impact of NG feeding on aspiration pneumonia | NGT significantly increased of developing aspiration pneumonia RR 12.609 (CI 95% OR 21.54), p<0.0001 |

aOR – adjusted Odds Ratio, aHR – adjusted Hazard Ratio, aRR – adjusted Relative Risk, CI – Confidence Interval, HLA-DR - Human leukocyte antigen DR, IQR – Inter quartile range, MDT – Multidisciplinary Team, NBM – Nil by Mouth, NGT – Nasogastric Tube, NIHSS – National Institutes of Health Stroke Scale, OR – Odds Ratio, PPI – Proton Pump Inhibitors, RR – Relative Risk, SAP – Stroke-associated pneumonia, SD – Standard Deviation, SDD – selective decontamination of the digestive tract , RR – Relative Risk

Table 4: Quality Appraisal Tables

Appraisal Tool: Cochrane Collaboration’s tool for assessing risk of bias

| **Study** | **Domain** | **Description** | **High Risk of Bias** | **Low Risk of Bias** | **Unclear Risk of Bias** | **Reviewer Assessment** | **Reviewer Comments** |
| --- | --- | --- | --- | --- | --- | --- | --- |
| **Gosney et al. (2006)** | **Selection bias** – Random sequence generation | Computer-generated random numbers |  | Low |  | Low |  |
|  | **Selection bias** – Allocation Concealment | Not described |  |  | Not described | Unclear | Random sequence undertaken by the Research Pharmacist |
|  | **Performance bias** – Blinding (participants and personnel) | Double blind – participants and researchers |  | Low |  | Low |  |
|  | **Detection bias** – Blinding (outcome assessment) | Double blind – participants and researchers |  | Low |  | Low |  |
|  | **Attrition bias** – Incomplete outcome data | Completeness of outcome data reported |  | Low |  | Low |  |
|  | **Reporting bias** – Selective reporting |  |  |  | Unclear | Unclear | Unclear if there was a published protocol or trial registered. |
|  | **Other bias** – Other sources of bias | Insufficient information to assess whether an important risk of bias exists. |  |  | Unclear | Unclear |  |

| **Study** | **Domain** | **Description** | **High Risk of Bias** | **Low Risk of Bias** | **Unclear Risk of Bias** | **Reviewer Assessment** | **Reviewer Comments** |
| --- | --- | --- | --- | --- | --- | --- | --- |
| **Kalra et al. (2015), UK** | **Selection bias** – Random sequence generation | Computer-generated |  | Low |  | Low | Selection bias in cluster-randomized trial could result from patients at increased risk of SAP being recruited preferentially to the antibiotic intervention group. |
|  | **Selection bias** – Allocation Concealment | Patients, research staff obtaining data, and statisticians undertaking analyses of the outcome data unaware of stroke unit allocation. |  | Low |  | Low |  |
|  | **Performance bias** – Blinding (participants and personnel) | Open label trial | High |  |  | High | Participants and researchers aware of allocation of treatment. Open-intervention allocation can influence physician diagnosis of SAP. |
|  | **Detection bias** – Blinding (outcome assessment) | Minimized by criteria based algorithm for diagnosis of SAP, applied blind to the whole dataset. Outcomes assessed by researchers marked to allocation. |  | Low |  | Low | Algorithm missed a diagnosis of SAP in 10% of patients. |
|  | **Attrition bias** – Incomplete outcome data | Completeness of outcome data reported |  | Low |  | Low |  |
|  | **Reporting bias** – Selective reporting | Reporting of primary and secondary outcome measures |  | Low |  | Low | Primary and secondary outcome measures reported consistent with registered trial isrctn.com, number ISRCTN37118456 |
|  | **Other bias** – Other sources of bias |  |  |  | Unclear | Unclear |  |

| **Study** | **Domain** | **Description** | **High Risk of Bias** | **Low Risk of Bias** | **Unclear Risk of Bias** | **Reviewer Assessment** | **Reviewer Comments** |
| --- | --- | --- | --- | --- | --- | --- | --- |
| **Kalra et al. (2016), UK** | **Selection bias** – Random sequence generation | Computer-generated |  | Low |  | Low | Selection bias in cluster-randomized trial could result from patients at increased risk of SAP being recruited preferentially to the antibiotic intervention group. Inclusion criteria for RCT (Kalra et al 2015) may have resulted in selection bias. |
|  | **Selection bias** – Allocation Concealment | Patients, research staff obtaining data, and statisticians undertaking analyses of the outcome data unaware of stroke unit allocation. |  | Low |  | Low |  |
|  | **Performance bias** – Blinding (participants and personnel) | Open label trial | High |  |  | High | Participants and researchers aware of allocation of treatment. Open-intervention allocation can influence physician diagnosis of SAP. |
|  | **Detection bias** – Blinding (outcome assessment) | Minimized by criteria based algorithm for diagnosis of SAP, applied blind to the whole dataset. Outcomes assessed by researchers marked to allocation. |  | Low |  | Low | Algorithm missed a diagnosis of SAP in 10% of patients. |
|  | **Attrition bias** – Incomplete outcome data | Completeness of outcome data reported. Analysis done on an intention to treat basis. |  | Low |  | Low | Bias from missing data was reduced by the use of multiple imputation assuming missingness at random. |
|  | **Reporting bias** – Selective reporting | Reporting of primary and secondary outcome measures |  | Low |  | Low | Primary outcome measure reported consistent with registered trial isrctn.com, number ISRCTN37118456. NGT status included as additional secondary outcome in paper. |
|  | **Other bias** – Other sources of bias |  |  |  | Unclear | Unclear |  |

| **Study** | **Domain** | **Description** | **High Risk of Bias** | **Low Risk of Bias** | **Unclear Risk of Bias** | **Reviewer Assessment** | **Reviewer Comments** |
| --- | --- | --- | --- | --- | --- | --- | --- |
| **Warusevitaine et al. (2014)** | **Selection bias** – Random sequence generation | Random numbers list generated by an independent statistician placed in a opaque-sealed envelope |  | Low |  | Low |  |
|  | **Selection bias** – Allocation Concealment | Once consent obtained a staff member not involved in the study released the envelope. |  | Low |  | Low |  |
|  | **Performance bias** – Blinding (participants and personnel) | Nurse administering the drug was not blinded to treatment. The researcher and medical team involved in the patient’s care were blinded to treatment. |  | Low |  | Low | . |
|  | **Detection bias** – Blinding (outcome assessment) | Diagnosis of pneumonia done by an independent clinician. |  | Low |  | Low |  |
|  | **Attrition bias** – Incomplete outcome data | Completeness of outcome data reported. |  | Low |  | Low |  |
|  | **Reporting bias** – Selective reporting | Reporting of primary and secondary outcome measures |  | Low |  | Low | Primary outcome measure reported consistent with registered trial isrctn.com, number ISRCTN18034911. Did not report secondary outcome number of different antibiotics used. |
|  | **Other bias** – Other sources of bias |  |  |  | Unclear | Unclear |  |

Appraisal Tool: CASP Cohort Study Checklist

| **Aoki et al. (2016)**  Section (A) Are the results of the study valid? | (1) Did the study address a clearly focused issue?  (2) Was the cohort recruited in an acceptable way? (Risk of selection bias)  (3) Was the exposure accurately measured to minimise bias? (risk of measurement or classification bias)  (4) Was the outcome accurately measured to minimise bias?  (5a.) Have the authors identified all important confounding factors?  (5b.) Have they taken account of the confounding factors in the design and/or analysis?  (6a.) Was the follow up of subjects complete enough?  (6b.) Was the follow up of subjects long enough? | Yes – Influence of a multidisciplinary team approach to dysphagia on rates of SAP prior period and post period team organization  Yes – All consecutive stroke patients admitted April 2009 and March 2014. The cohort was representative of the clinical population of interest.  Yes – The exposure was the introduction of MDT participatory swallowing team. There was a defined prior period before team organization and post period after team organization.  Yes – See Table 4. SAP was diagnosed according to CDC Criteria.  Yes – Groups did not differ significantly on age, sex, vascular risk factors, NIHSS score on admission and stroke sub type  Yes – The people assessing the outcomes were blinded to the period of admission.  Yes  Yes for the purposes of SAP. |
| --- | --- | --- |
| Section (B) What are the results? | (7) What are the results of the study?  (8) How precise are the results?  (9) Do you believe the results? | See Table 3-4  Results appear precise and are statistically significant.  Yes. |
| Section C - Will the results help locally? | (10) Can the results be applied to the local population?  (11) Do the results of this study fit with other available evidence?  (12) What are the implications of this study for practice? | Yes – the results are clinically relevant.  Yes – In line with Gandolfi et al.  The results suggest a multidisciplinary swallowing team approach which includes increased oral care and instrumental assessment reduces pneumonia onset |

| **Arai et al. (2017)**  Section (A) Are the results of the study valid? | (1) Did the study address a clearly focused issue?  (2) Was the cohort recruited in an acceptable way? (Risk of selection bias)  (3) Was the exposure accurately measured to minimise bias? (risk of measurement or classification bias)  (4) Was the outcome accurately measured to minimise bias?  (5a.) Have the authors identified all important confounding factors?  (5b.) Have they taken account of the confounding factors in the design and/or analysis?  (6a.) Was the follow up of subjects complete enough?  (6b.) Was the follow up of subjects long enough? | Yes - To assess whether acid-suppressive drugs increase pneumonia in acute stroke.  Yes – Acute stroke patients admitted from 1 January 2006 to 1 January 2016. Does not explicitly state consecutive cohort. However, clear description of patient selection and declaration of ineligible patients from the total sample. To minimize confounding with respect to risk factors of pneumonia, only those could not feed orally (enteral feeding was permitted) for 14 days> p.a. Data was retrospectively collected.  Yes – Exposure to H2B and PPI was confirmed by reading medical charts. Each patient’s daily exposure and days at risk were recorded. Of patient’s exposure status changed (e.g. H2B to PPI), days at risk were measured separately. Additional analysis performed of subjects excluding those receiving both drugs.  Yes – See Table 4. SAP was diagnosed according to CDC Criteria.  Yes - The authors attempted to control with respect to risk of pneumonia, exposure to acid suppressive drugs, and antibiotics administered for other infections.  Yes – Multivariate Poisson regression and propensity score analysis performed to adjust for possible confounders.  Yes  Yes for the purposes of SAP. |
| --- | --- | --- |
| Section (B) What are the results? | (7) What are the results of the study?  (8) How precise are the results?  (9) Do you believe the results? | See Table 3-4  Results appear precise and are statistically significant.  Yes |
| Section C - Will the results help locally? | (10) Can the results be applied to the local population?  (11) Do the results of this study fit with other available evidence?  (12) What are the implications of this study for practice? | Yes – the results are clinically relevant.  Yes – In line with Herzig et al [14].  Suggests that prophylactic acid suppressive therapy with PPI may have to be avoided in acute stroke patients susceptible to pneumonia. |

| **Brogan et al. (2015)**  Section (A) Are the results of the study valid? | (1) Did the study address a clearly focused issue?  (2) Was the cohort recruited in an acceptable way? (Risk of selection bias)  (3) Was the exposure accurately measured to minimize bias? (risk of measurement or classification bias)  (4) Was the outcome accurately measured to minimize bias?  (5a.) Have the authors identified all important confounding factors?  (5b.) Have they taken account of the confounding factors in the design and/or analysis?  (6a.) Was the follow up of subjects complete enough?  (6b.) Was the follow up of subjects long enough? | Yes - To investigate which factors are most strongly associated with infections in acute stroke.  Yes – Review of medical records of patients with a primary admission diagnosis of stroke admitted to Australian tertiary hospitals admitted in 2010. Unclear if consecutive cohort but authors declare the reason and number of illegible patients from the sample.  Yes - Predictors of respiratory infection were defined. Potential for associations with risk of infection to reflect associations with severity and possibility that infection was present prior to stroke.  Unclear. Infections developed <7days p.a. No definition of respiratory infection was defined. Based on classification by medical and SLP staff.  Yes  Yes – Relationships between clinical factors and infections analysed using univariate and multivariate binary logistic.  Yes  Yes for the purposes of SAP. |
| --- | --- | --- |
| Section (B) What are the results? | (7) What are the results of the study?  (8) How precise are the results?  (9) Do you believe the results? | See Table 3-4  Results appear precise and are statistically significant.  Yes |
| Section C - Will the results help locally? | (10) Can the results be applied to the local population?  (11) Do the results of this study fit with other available evidence?  (12) What are the implications of this study for practice? | Yes – the results are clinically relevant.  Yes – Period of susceptibility to infection (Westendorp et al [4], Wartenberg et al. [6]) and multifactorial nature of SAP (Langmore et al. [17]).  Suggests that patients with severe dysphagia require NGT are significantly associated with infections developing <7 days post stroke. |

| **Gandolfi et al. (2014)**  Section (A) Are the results of the study valid? | (1) Did the study address a clearly focused issue?  (2) Was the cohort recruited in an acceptable way? (Risk of selection bias)  (3) Was the exposure accurately measured to minimise bias? (risk of measurement or classification bias)  (4) Was the outcome accurately measured to minimise bias?  (5a.) Have the authors identified all important confounding factors?  (5b.) Have they taken account of the confounding factors in the design and/or analysis?  (6a.) Was the follow up of subjects complete enough?  (6b.) Was the follow up of subjects long enough? | Yes - To evaluate the impact of standardized multidisciplinary protocol on clinical outcomes in patients with post stroke dysphagia.  Yes – Review of medical records of patients with post stroke dysphagia to the neurological ward of Verona University Hospital between January 2004 and December 2008. Unclear if consecutive cohort but clear declaration of numbers/reason for records excluded. Potential for selection bias in T+ group – although unadjusted and adjusted OR did not differ suggesting good data compatibility  Yes – Protocol consisted of two consecutive phases: diagnostic and rehabilitation. Clearly described.  Yes – See Table 4. Pneumonia determined by the presence of 3> of the following variables: fever (>38 °C), abnormal chest radiograph, productive cough with purulent sputum, abnormal respiratory examination, combined antibiotic therapy.  Yes – Adjusted for sex, age, previous CVA or TIA, dementia, dysarthria, type of stroke and barthel index on admission.  Yes – Differences between the T+ and T- group, effects of the standardized clinical protocol for dysphagia on clinical outcomes were statistically analyzed and aOR and 95% CI were calculated using logistical regression.  Yes  Yes for the purposes of SAP. |
| --- | --- | --- |
| Section (B) What are the results? | (7) What are the results of the study?  (8) How precise are the results?  (9) Do you believe the results? | See Table 3-4  Results appear precise and are statistically significant.  Yes |
| Section C - Will the results help locally? | (10) Can the results be applied to the local population?  (11) Do the results of this study fit with other available evidence?  (12) What are the implications of this study for practice? | Yes – the results are clinically relevant.  Yes – See Aoki et al.  Suggests treatment under a standardized protocol delivered by a MDT can significantly reduce aspiration pneumonia. |

| **Hoffman et al. (2016)**  Section (A) Are the results of the study valid? | (1) Did the study address a clearly focused issue?  (2) Was the cohort recruited in an acceptable way? (Risk of selection bias)  (3) Was the exposure accurately measured to minimize bias? (risk of measurement or classification bias)  (4) Was the outcome accurately measured to minimize bias?  (5a.) Have the authors identified all important confounding factors?  (5b.) Have they taken account of the confounding factors in the design and/or analysis?  (6a.) Was the follow up of subjects complete enough?  (6b.) Was the follow up of subjects long enough? | Yes - To confirm that markers of immunodepression predict SAP independently from dysphagia. To determine the diagnostic value of selected markers of immune function (mHLA-DR), inflammation (IL-6) and infection (LBP) on day 1 in improving prediction of SAP between 2 and 7 days after stroke onset.  Yes – Daily screening for eligible patients. All eligible patients were asked for participation.  Yes – Collection of immunological data clearly described.  Yes – See Table 4. Diagnosis of SAP during hospitalisation defined by the study/treating physician based on CDC criteria.  Yes – Baseline characteristics stratified by pneumonia.  Yes – To estimate Odds Ratio (OR) and resulting 95% CI of the association between quartiles of biomarkers and SAP risk, logistic regression analysis were performed. Analyses stratified by dysphagia status.  Yes  Yes for the purposes of SAP. |
| --- | --- | --- |
| Section (B) What are the results? | (7) What are the results of the study?  (8) How precise are the results?  (9) Do you believe the results? | See Table 3-4.  Results appear precise and are statistically significant.  Yes |
| Section (C) - Will the results help locally? | (10) Can the results be applied to the local population?  (11) Do the results of this study fit with other available evidence?  (12) What are the implications of this study for practice? | Yes – the results are clinically relevant.  Unclear – Proof of concept study designed to confirm that selected biomarkers are predictors for SAP independent of presence of dysphagia.  Suggests screening for immune markers and dysphagia allows for identification of patients at high risk of SAP. |

| **Langdon et al. (2009)**  Section (A) Are the results of the study valid? | (1) Did the study address a clearly focused issue?  (2) Was the cohort recruited in an acceptable way? (Risk of selection bias)  (3) Was the exposure accurately measured to minimize bias? (risk of measurement or classification bias)  (4) Was the outcome accurately measured to minimize bias?  (5a.) Have the authors identified all important confounding factors?  (5b.) Have they taken account of the confounding factors in the design and/or analysis?  (6a.) Was the follow up of subjects complete enough?  (6b.) Was the follow up of subjects long enough? | Yes – Whether patients who are NBM and tube fed have higher risk of developing infections due to aspiration of bacteria-laden saliva or refluxed material than stroke patients who are fed orally.  Unclear – Participants admitted between 1 January-30 September 2005. Does not explicitly state consecutive cohort. Declares number of participants approached and number who declined participation.  Yes – Subjects followed up 2-3 days to determine whether they had been diagnosed with infection. Chart of the cohort showing tube feeding and infection rates clearly explained.  Yes - See Table 4. Diagnosis of SAP within 30 days defined by the treating physician.  Results may be confounded in that NGTs are often used in patients with severe dysphagia, so the relationship between SAP and NGT may be more likely explained by dysphagia not NGT use.  Yes – Logistic regression analyses undertaken to ascertain relationship between clinical factor, respiratory infections, and all infections, with statistical significance set at 5%.  Yes  Yes for the purposes of SAP. |
| --- | --- | --- |
| Section (B) What are the results? | (7) What are the results of the study?  (8) How precise are the results?  (9) Do you believe the results? | See Table 3-4.  Results were statistically significant.  Yes |
| Section (C) - Will the results help locally? | (10) Can the results be applied to the local population?  (11) Do the results of this study fit with other available evidence?  (12) What are the implications of this study for practice? | Yes – the results are clinically relevant.  Period of susceptibility to infection (Westendorp et al [4], Wartenberg et al. [6]) and association of NGT with respiratory infections re. Brogan.  Suggests patients NBM patients with NGT have significantly higher rates of respiratory infection than those fed orally. However potential for confounding (see above). |

| **Schwarz et al. (2017)**  Section (A) Are the results of the study valid? | (1) Did the study address a clearly focused issue?  (2) Was the cohort recruited in an acceptable way? (Risk of selection bias)  (3) Was the exposure accurately measured to minimize bias? (risk of measurement or classification bias)  (4) Was the outcome accurately measured to minimize bias?  (5a.) Have the authors identified all important confounding factors?  (5b.) Have they taken account of the confounding factors in the design and/or analysis?  (6a.) Was the follow up of subjects complete enough?  (6b.) Was the follow up of subjects long enough? | Yes – To determine the impact of aspiration pneumonia and NGT feeding on patient outcomes, cost and length of stay.  Potential risk of selection bias due to purposeful sample of medical notes of patients admitted between January 2011-December 2014.  No formalized or standardized assessment tools were used.  Unclear – See Table 5  No. Authors identify limitations of retrospective chart auditing, small sample size, and auditing by one author. Other potential confounders such as NIHSS, severity of dysphagia, unaccounted for.  Partially. Data analysis included parametric and non parametric methods however these were not adjusted for potential confounding factors.  Yes  Unclear. |
| --- | --- | --- |
| Section (B) What are the results? | (7) What are the results of the study?  (8) How precise are the results?  (9) Do you believe the results? | See Table 3-4.  Only provided descriptive statistics for SAP. 95% CI provided for cost and length of stay.  Yes – Incidence of pneumonia concurred with Kishmore et al [1] |
| Section (C) - Will the results help locally? | (10) Can the results be applied to the local population?  (11) Do the results of this study fit with other available evidence?  (12) What are the implications of this study for practice? | Yes – the results are clinically relevant.  Yes – post stroke pneumonia is associated with increased mortality, LOS and associated healthcare costs.  Supports early identification, assessment and management of dysphagia to minimize risk of post stroke pneumonia. |

Table 5: Criteria for SAP, period of diagnosis and incidence

| Author, year, county | Criteria for diagnosis | Period of diagnosis | Incidence |
| --- | --- | --- | --- |
| Aoki et al. (2016), Japan | Centre for Disease Control and Prevention (Harms et al.) Reference 10:  Presence of a new and persistent infiltrate or consolidation on at least 1 chest X-ray or CT with one of the following clinical signs: fever, leukopenia or leukocytosis and altered mental status in more than 70 yr. olds in the absence of other causes. These should be added to 2 of the following signs: new-onset purulent sputum or change in character of the sputum, new onset or progressive cough, rales, and impaired gas exchange. | During hospitalization | SAP prior period 15.9% (N=21) vs. 6.9% (N=12) post period; p=0.01. |
| Arai et al. (2017), Japan | Centre for Disease Control and Prevention (Garner et. Al) Reference 24. | Within 2 weeks after admission | SAP 39.4% (N=132) (95% CI; 34.2-44.9%) |
| Brogan et al. (2015), Australia | No definition provided for “respiratory infection”. | First 7 days from admission | SAP 11.26% (N=60) |
| Gandolfi et al. (2014), Italy | Presence of 3>: fever (>38 °C), abnormal chest radiograph, productive cough with purulent sputum, abnormal respiratory examination, combined antibiotic therapy | During hospitalisation | SAP no significant differences between the two groups in frequency of pneumonia (% not provided). SAP aOR 0.34 (0.07-1.49) |
| Gosney et al. (2006), UK | Clinical signs and symptoms of pneumonia as recorded in the case notes | During hospitalization | SAP 3.9% (N=8/203) |
| Hoffman et al. (2016), Germany | Defined by the study physician based on clinical symptoms and/or radiological findings and/or pathogen detection of pulmonary infection. Criteria for ‘clinically defined pneumonia’ by the US Centers for Disease Control and Prevention (CDC) were assessed at each visit. | During hospitalization | SAP 5.2% (N=25/479) |
| Kalra et al. (2015), UK | Blinded application of Centers for Disease Control and Prevention criteria for pneumonia (Horan et al.) Reference 14, that interrogated 8 clinical/laboratory observations at the 6 recorded time points in the whole patient group for temperature ≥37.5°C on 2 consecutive measurements or a single measurement of ≥38.0°C; respiration rate of ≥20 breaths per minute, cough and breathlessness, purulent sputum; and white cell count >11.0 x 109/L, chest infection on X Ray, positive sputum culture/microbiology, or positive blood culture.  Physician diagnosed SAP | In the first 14 days | Algorithm defined SAP 11.3% (N=123/1088).  Physician diagnosed SAP 15.8% (N=192/1217).  Algorithm defined SAP in antibiotics group 13% (N=71/564) vs. 10% (N=52/524) in control group; aOR 1.21 [95% CI 0.71-2.08], p=0.489, intraclass correlation coefficient [ICC] 0.06 [95% CI 0.02-0.17].  Physician diagnosed SAP 16% (N=101/615) vs. 15% (N=91/602), aOR 1.01 [95% CI 0.61-1.68], p=0.957, ICC 0.08 [95% CI 0.03-0.21]. |
| Kalra et al. (2016), UK | Blinded application of Centers for Disease Control and Prevention criteria for pneumonia (Horan et al.) Reference 16, that interrogated 8 clinical/laboratory observations at the 6 recorded time points in the whole patient group for temperature ≥37.5°C on 2 consecutive measurements or a single measurement of ≥38.0°C; respiration rate of ≥20 breaths per minute, cough and breathlessness, purulent sputum; and white cell count >11.0 x 109/L, chest infection on X Ray, positive sputum culture/microbiology, or positive blood culture.  Physician diagnosed SAP | In the first 14 days | Algorithm defined SAP 11.3% (N=123/1088).  Physician diagnosed SAP 15.8% (N=192/1217).  Algorithm defined SAP NGT 14.4% (N=43/298) vs. no NGT 10.1% (N=80/790); aOR 1.26 [95% CI 0.78-2.03], p=0.35.  Physician defined SAP NGT 18.5 (N=54/298) vs. no NGT 15.3 (n=138/919); 1.27 [95% CI 0.85-1.90), p=0.248. |
| Langdon et al. (2009) | Diagnosed by the attending clinician and based on the presence of 3>: fever ≥38°C, productive cough with purulent sputum, abnormal respiratory examination (tachypnea, > 22/min, tachycardia, inspiratory crackles, bronchial breathing), abnormal chest radiography, arterial hypoxemia (PO_2_ <70mm Hg), and isolation of a relevant pathogen (positive Gram stain and culture). | First 30 days after stroke | 15.5% SAP (N=51/330). SAP in NGT 40.5% (N=30/74) vs. 8.2% (N=21/256) fed orally, 73% SAP (N=22/30) in NGT patients diagnosed days 2-4 post stroke. |
| Schwarz et al. (2017) | Not reported | Not reported | 11.8% (N=13) aspiration pneumonia. |
| Warusevitaine et al. (2014) | Full clinical examination of the chest.  Inflammatory markers, sputum cultures, and chest radiographs were requested if there was a clinical suspicion of pneumonia. The diagnosis of pneumonia was made according to the British Thoracic Society recommendations, with minor modifications | 21 days | 56.7% SAP (N=34/60), 94% SAP within 7 days from admission.  26.7% SAP (N=8/30) Metoclopramide vs. 86.7% SAP (N=26/30) Placebo. |

Figure 2 – Overall rate of SAP

| **Study** | **Sample Size** | **% diagnosed with SAP (95% CI)** | **Random effects weights** | **% diagnosed with SAP, random effects, 95% CI** |
| --- | --- | --- | --- | --- |

| Arai et al. (2017) | 335 | 39.4% | (32.7, 46.1) | 10.5% |
| --- | --- | --- | --- | --- |
| Kalra et al. (2015, 2016) | 1088 | 11.3% | (9.3, 13.3) | 12.8% |
| Warusevitaine et al. (2014) | 60 | 56.7% | (37.6, 75.7) | 4.4% |
| Gosney et al. (2006) | 203 | 3.9% | (1.2, 6.7) | 12.5% |
| Hoffman et al. (2006) | 479 | 5.2% | (3.2, 7.3) | 12.7% |
| Aoki et al. (2016) | 173 | 6.9% | (3.0, 10.9) | 12.0% |
| Brogan et al. (2015) | 533 | 11.3% | (8.4, 14.1) | 12.5% |
| Langdon et al. (2009) | 330 | 15.5% | (11.2, 19.7) | 11.9% |
| Schwarz et al. (2017) | 110 | 11.8% | (5.4, 18.2) | 10.7% |

Random effects model: I^2^ = 72%; Q = 28.2 df = 8 (P <0.001)

Missing data for Gandolfi et al. (2014)

Studies stratified on axis by population: in order of dysphagia only, dysphagia vs. non-dysphagia and unselected patients.

In pre/post trials – post data %’ s reported.
